# Specialty Care Use in US Patients with Chronic Diseases

**DOI:** 10.3390/ijerph7030975

**Published:** 2010-03-10

**Authors:** Jessica D Bellinger, Rahnuma M Hassan, Patrick A Rivers, Qiang Cheng, Edith Williams, Saundra H Glover

**Affiliations:** 1 SC Rural Health Research Center, University of South Carolina, 800 Sumter Street HESC, 312B, Columbia, SC 29210, USA; 2 Institute for Partnership to Eliminate Health Disparities, University of South Carolina, 220 Stoneridge Drive Columbia, SC 29210, USA; E-Mail: hassanr@mailbox.sc.edu; 3 College of Applied Sciences and Arts, Health Care Management, Southern Illinois University Carbondale, 1365 Douglas Drive, MC 6615, Carbondale, IL 62901-6615, USA; E-Mail: privers@siu.edu; 4 Computer Science Department, Southern Illinois University Carbondale, 1000 Faner Drive, Faner 2125 MC 4511, Carbondale, IL 62901-6615, USA; E-Mail: qcheng@cs.siu.edu; 5 Institute for Partnership to Eliminate Health Disparities, University of South Carolina, 220 Stoneridge Drive Columbia, SC 29210, USA; E-Mails: willi425@mailbox.sc.edu (E.W.); sglover@sc.edu (S.H.G.)

**Keywords:** specialty care, health disparities, chronic disease

## Abstract

Despite efforts to eliminate health disparities, racial, ethnic, and geographic groups continue lag behind their counterparts in health outcomes in the United States. The purpose of this study is to determine variation in specialty care utilization by chronic disease status. Data were extracted from the Commonwealth Fund 2006 Health Care Quality Survey (n = 2475). A stratified minority sample design was employed to ensure a representative sample. Logistic regression was used in analyses to predict specialty care utilization in the sample. Poor perceived health, minority status, and lack of insurance was associated with reduced specialty care use and chronic disease diagnosis.

## 1. Introduction

The effort to eliminate racial and ethnic health disparities is one of the main goals of Healthy People 2010 [1]. Experts have studied various reasons for poor minority health outcomes and predisposition to certain diseases, such as genetic disposition, differences in cultural and nutritional practices, and a history of discriminatory health care in the United States [1,2]. Existing research explores patient level-characteristics (race/ethnicity, health behaviors, and perceived health status) and system-level characteristics, such as the availability of providers, access to health care institutions, or the characteristics of different types of providers. The purpose of this study is to determine rural/urban variation in specialty care utilization by perceived health and chronic disease status. The specific research aims are to determine if there are variations specialty care utilization based on (a) chronic disease status and (b) perceived health status.

### 1.1. Specialty Care and Primary Care

According to the American Medical Association, 40.4% of the practicing physicians in the US were primary care providers in 2005 compared to 59.6% of specialists [3]. The largest subspecialties were surgical specialties (14.6%) of the specialists and internal physicians (15.1%) of primary care providers [3]. The characteristics of providers have been posited as sources for the disparities health outcomes reported in certain populations and a potential source of the health disparities prioritized in the national research agenda in Healthy People 2010.

The effectiveness of specialty care compared to primary care has been studied extensively in health services research literature. Specialists, those that practice a subspecialty of internal medicine, receive continued postgraduate medical training [4]. A seminal review of the knowledge and quality of care comparing specialists and generalists suggests specialists are better equipped to handle patients with specific conditions, such as myocardial infarction, depression, and HIV/AIDS; however, specialists are also more likely to overuse unnecessary diagnostic therapies that increase the cost of care [4]. In contrast, generalists are more knowledgeable about a wider range of diseases and more equipped for health promotion and disease prevention [4].

The comparison of specialists and generalist can be a contentious and acrimonious exercise with mixed findings or may contribute to professional discord [5]. It has been suggested that such comparisons are not helpful and negate the team-based approach necessary for the provision of quality health care [2]. A strong partnership with complementary roles for specialists and primary care providers has been suggested as a more appropriate framework for a discussion of the different types of care available to patients in the United States. In fact, recent literature suggests that shared responsibility of care delivers a better quality of care than either primary or specialty care alone, especially for patients with chronic disease [6,7].

It has also been noted that shared care or the “joint participation of…practitioners in the planned delivery of care for patients with a chronic condition” allows a more efficient use of limited specialty resources [7]. In an era of soaring health care costs and declining resource, debate remains whether primary care can serve as a substitute or complement specialty services. While a Department of Veteran Affairs study demonstrated primary care can serve as a substitute while not increasing the costs of care, these findings are not generalizable to the population at large or for people with chronic diseases [8]. Proponents of shared care would contend that primary care serves as a complement for specialty services by providing the benefits and expertise of specialists combined with the continuity of care provided by generalists [7,9–11].

### 1.2. Access to Specialty Care

Studies suggest minorities are less likely to receive specialty care, but access to specialty care is mediated by various factors, such as physician referral, geographic location, and insurance type. Studies have also shown that residents of rural areas are less likely to have access to health care providers, health insurance and specialty care. As a result they are more likely to experience health care disparities and perceive their health as poorer than their counterparts [4]. Clancy and Franks reported that males, patients with HMOs, and those with longer visit lengths were more likely to be referred by a primary care physician [12]. Patients with Medicaid and Medicare also had difficulties accessing specialty care [5]. Low income populations are also less likely to access specialty care [6]. However, some patients avoid the barriers of the gate-keeping system to specialty care by self-referral. Findings from the National Ambulatory Care Survey suggest that African Americans, Medicaid beneficiaries, women, and HMO patients are less likely to refer themselves to specialty care [12].

Rural patients were also significantly less likely to seek specialty care for a problem without physician referral. Patients with more chronic diseases were seen by primary care physicians than specialists, but more patients with neurologic disorders, psychiatric disorders, and cancer as the main diagnostic categories were seen by specialists through self-referral [13]. The increased enrollment in managed care organizations and policies restricting access to specialists has led researchers to examine referrals from primary care physicians to specialists [14–16]. Clancy and Franks (1997) performed a study and concluded an increased likelihood of being referred was associated with being a male patient. The regression analyses for the study were adjusted for patient factors such as age, race, sex, insurance and diagnostic category. Physician factors included age, sex, specialty and degree of specialization and practice factors such as rural location, region and proportion of HMO patients [14]. There is a need to increase health equity by creating access for patients who are unable to utilize services because of their geographic location. Equity is defined as “the absence of systematic disparities in health between social groups who have different levels of underlying social advantage” [15]. In a study using from the Medical Expenditure Panel Survey to determine the estimate of Americans who access different types of primary care and specialty physicians. It was concluded family physicians were the most accessed group and there were no income disparities in access [15].

Satisfaction and health behaviors serve mediating roles in patients realizing access to specialty care. Primary provider satisfaction with specialty care was found to be associated with specialist follow-up for differential diagnoses, management suggestions, and cohesive management plans [17]. However, a cross-sectional study of the factors influencing the selection of specialists by patients found patient convenience and good communication were major factors [18]. In response to the perception of non-compliant patient behaviors among minorities, African-American providers were more likely than white providers to consider patient convenience when choosing a specialist [18]. In addition, African Americans have been found to be less trusting of specialty care than white patients [19]. Patients with multiple sclerosis reported low satisfaction with interpersonal and access components of specialty care, but primary care rated low on treatment plans. It has been suggested that managed care efforts to regulate access to specialists and reduce cost has the potential to lower quality of care; additionally, few studies have examined the impact of managed care on patient perceptions of quality of care provided by physician and non physician specialist [20].

Overall, access to specialty care is complex and research suggests a shared or complementary role is optimal [7]. However, patients with multifactorial diseases require an additional level of expertise other than solely primary care, which is often best provided by specialists. Andersen’s access model provides a framework and guidelines to better understand the utilization of health services by those who are considered members of vulnerable populations. Vulnerable populations include minorities, mentally ill and chronically disabled persons. Andersen’s Behavioral Model for Vulnerable Populations “includes domains relevant to understanding the health and health seeking behavior of vulnerable population” [9]. Applying the integrated approaches suggested by the model can be helpful in determining what challenges members of vulnerable populations encounter when trying to access and utilize specialty care [21].

### 1.3. Specialty Care and Chronic Disease

Mental health services are an essential component of quality health care, however disparities in access and quality place minorities at risk for poor mental health [9]. Poor minorities (African Americans and Hispanics) were less likely to receive specialty mental health services when controlling for poverty levels [22]. Treatment of asthma was also found to be poor for both providers and patients in a survey of insured people in several regions throughout the region [23]. Typically, patients over utilized short-term relief measures and did not adhere to long-term control of asthma while providers were remiss in the accurate diagnosis of the disorder. Specialists had higher compliance rates with the national guidelines, indicating a better quality of care for their patients [24]. This trend continues in other studies which have also determined specialist care improves the quality of care through better asthma control [25–27].

Several studies have indicated specialty diabetes care is associated with better health outcomes for patients [28–31]. Given the limitations of the measures used, Shah and colleagues attempted to hone in on glycemic control, a diabetes-specific measure. Patients treated by specialists had lower A1C levels than patients treated by primary care providers [25,32]. Shah suggests the early initiation of insulin treatment by specialists was a factor in the lower A1C levels [32]. The findings of a recent population-based cohort study in Canada are mixed in regards to specialty care and diabetes. McAlister and colleagues report specialty care improved disease-specific measures, such as appropriate prescription drug use and insulin use [33].

The findings about the relative effectiveness of specialty care for patients with cardiovascular disease are mixed [32–38]. Studies using Medicare claims data identified a mortality differential after acute myocardial infarctions based on physician specialty [34,35]. In a study evaluating heath outcomes after acute myocardial infarctions based on the specialty of the admitting physician, Jollis and colleagues reported a significant decrease in 1-year mortality rates for Medicare beneficiaries treated by cardiologists [32]. The increased survival rate was accompanied by increased costs from higher rates of cardiac treatments and medications [34]. Given the critical role of specialist in providing quality care to patients with chronic diseases, the purpose of the study is to examine specialty care use in people with self-reported chronic disease.

## 2. Methods

The Commonwealth Fund’s 2006 Health Care Quality Survey is a nationally-representative telephone survey of adults living in the United States that measures health care utilization and quality of care. The survey gathers information about self-reported health status, perceived discrimination in the healthcare setting, patient preference, and other measures to allow for the effect of these satisfaction/quality measures on health care utilization. The survey, administered during the spring and fall of 2006, has an adult sample of 3,535 respondents (1,153 Hispanics, 1,037 African Americans, and 621 Asian Americans). A stratified minority sample design was employed to ensure a representative sample of minorities. To reduce loss of information and account for missing data, respondents with missing values from relevant items were not included in our final sample (n = 2,475).

To explore the important factors and relationship for the utilization of specialty care, we used specialty care utilization as a dependent variable defined as whether respondents had seen a specialist for a particular health problem two years prior to interview. The primary outcome variable was dichotomized into no specialty care visits (*i.e.*, 0) and one or more specialty care visits (*i.e.*, 1). Other variables included a regular health care provider or regular source of care. A secondary dependent variable modeled is chronic disease status defined as a self-report of any of the following chronic diseases: diabetes, hypertension, cardiovascular disease, and/or lung disease.

Other variables included are predisposing, enabling, and need categories defined by our theoretical framework. Predisposing characteristics of interest include age (18–44, 45–64, and over 65 years), sex, nativity status (foreign-born or US-born), education, employment, and marital status. Enabling variables were health insurance coverage and regular health care provider. Health insurance categories are insured with prescription coverage, insured without prescription coverage, and uninsured. Perceived health status measured the need for care.

### Data Analysis

Bivariate and multivariate analyses were used to predict specialty care utilization by selected variables. Logistic regression and chi-square analysis were used in the analysis of the data. The outcome variable is utilization of specialty care. We modeled the relationship between the utilization of specialty care and explanatory variables identified by our theoretical framework. In addition, chronic disease status (aggregate of self-reported chronic disease) was modeled using logistic regression appropriately weighted for survey estimates. SAS 9.2 was used to perform all analyses [39].

Initially, utilization of specialty care was modeled against sixteen predictor variables: language of interview; Hispanic ethnicity; perceived health, race, regular health care provider, rurality, age, education, employment, income, insurance, marital status, nativity status, health insurance, sex, and chronic disease status. The statistically significant variables were retained and reported from this full model. A similar approach was employed to model predictors of chronic disease status. The theoretical framework and statistical significance at 0.05 guided the inclusion of select predictors in the final model: sex, race, perceived health, age insurance, regular health care provider, income and nativity.

## 3. Results

### 3.1. Sample Characteristics

The study sample (n = 2,475) was very diverse: whites (43%), African Americans (32%), and Others (25%). The sample was overwhelmingly urban (87.3%) especially for minorities; whites reported the largest number of rural respondents, 18.7% (Table 1).

There were more women in the sample (67.4%) than men. The majority of the adults were younger than 44 years old and approximately a quarter were between 45 and 64 years of age (Table 1). All of the minority groups in the sample were younger than whites. While less than 40% of whites were younger than 44 years of age, more minority respondents were between the ages of 18−44, African Americans (43%) and Others (62%). The majority of respondents (53%) self-identified as married; however, only 38% of the African-American respondents were married (Table 1). Most respondents were US-born (76%) and completed the interview in English (94%). However, over 56% of respondents classified as Others (including Asians and Hispanics) were foreign-born and 3% of completed the interview in a language other than English.

Approximately 30% of the sample reported a high school diploma or GED; however, less than a third of African Americans earned a high school diploma (Table 1). Overall, one in five respondents reported being unemployed at the time of interview. More African Americans reported poor health (23%) than whites (20%).

In addition to demographic characteristics, the ability of respondents to access the health system was measured. Less than 9% of the respondents were uninsured. Over 81% of the respondents were insured and 9% reported health insurance, but lacked prescription coverage. The majority (83%) reported no interruptions in their health insurance coverage in the year prior to the interview (Table 1). In addition to health insurance, most (90%) reported a regular primary care provider (Table 1).

Chronic disease status was measured with 46% of the sample reporting at least one chronic disease; however, over 56% of African Americans reported a chronic disease (Table 2). Nearly a third of the sample reported being told by a doctor of a hypertension diagnosis, 42% of African Americans reported hypertension (Table 2). While 17% of African Americans reported diabetes, less than 13% of the total sample reported diabetes. Less than 9% of the sample reported any heart disease. In contrast, less than 4% of the Others reported cardiovascular disease (Table 2).

### 3.2. Specialty Care Utilization

Table 3 describes the predictors of specialty care utilization for the full model and final model. Race, age, employment, language of interview, access, and perceived health and chronic disease status were significant predictors of specialty care utilization in the full model. In the final model, the adjusted odds ratios (AOR) are presented when controlling for race/ethnicity, gender, age, income, insurance, regular health care provider and perceived health status as indicated. African Americans were less likely to report specialty care than whites (OR = 0.7, 95% CI = 0.52, 0.81; AOR = 0.81, 95% CI = 0.67, 0.99). Compared to the youngest respondents (18−44 years old), the oldest respondents were more likely to report specialty care (AOR = 1.76, 95% CI = 1.35, 2.31; Table 3).

Low-income (less than $25,000 annually) was associated with specialty care use when compared to those making more than $60,000 annually (AOR = 1.43, 95% CI = 1.10, 1.85). Respondents lacking health insurance were less likely to report specialty care use (AOR = 0.42, 95% CI = 0.28, 0.61). Those with health insurance, but lacking prescription drug coverage were also less likely to report specialty care use (AOR = 0.59, 95% CI = 0.42, 0.85; Table 3). As a measure of specialty care need, those with reported poor/fair health had markedly higher odds of using specialty care than those with good/excellent perceived health (AOR = 1.74, 95% CI = 1.33, 2.23; Table 3.)

Table 4 outlines the predictors of chronic disease status, the secondary outcome, for both the full and final model. In the final model, the adjusted odds ratios (AOR) are presented when controlling for specific variables as noted. Race/ethnicity, age, nativity status, access, and perceived health were predictors of chronic disease status. African Americans were more likely to report at least one chronic disease than whites (AOR = 1.66, 95% CI = 1.33, 2.08; Table 4). Older respondents had markedly higher odds of reporting a chronic disease than those in the youngest age group, 18−44 years old. Middle-aged respondents (45−64) were more likely to report a chronic disease than the youngest group (AOR = 3.90, 95% CI = 3.07, 4.95). Those born in a foreign country were less likely to report a chronic disease than respondents born in the United States (AOR = 0.55, 95% CI = 0.42, 0.72). Lack of a usual source of care was a negative predictor of chronic disease status (AOR = 0.47, 95% CI = 0.31, 0.70; Table 4).

## 4. Conclusions

The Commonwealth Fund’s survey measures health care utilization and quality of care. However, certain factors were associated with increased utilization and poor health outcomes measured by chronic disease status. Overall, perceived need (as measured by self-report of health), race/ethnicity, access, and age were predictors of both specialty care use and chronic disease status. Similarly to previous studies, perceived poor health and older age are associated with health care utilization and poorer health outcomes, such as a chronic disease diagnosis [40–42].

Half of the sample reported specialty care utilization in a relatively low-income sample; over 70% of the respondents earned less than $25,000 annually (Table 2). In fact, low income respondents were more likely to report specialty care use (Table 3), which suggests that public health insurance programs for low-income Americans provide sufficient access to care.

In a discussion of health disparities and health care utilization, African Americans have poorer health outcomes and typically impeded access to the health system [40–44]. We found African Americans had the highest level of chronic disease (56%) compared to all other respondents. African Americans were less likely to report specialty care use and more likely to report a chronic disease. This finding suggests an opportunity to educate this population about the appropriateness of specialty care, especially with a chronic disease.

## 5. Discussion

This study highlights the need for health disparities research, especially among vulnerable groups. African Americans in the sample reported higher rates of chronic disease, which puts them at risk for poor health outcomes and a greater need for specialty care; however, African Americans were less likely to report specialty care use. Such findings represent an opportunity to work towards the elimination of health disparities. System-level barriers, such as the availability of providers (both primary and specialty), could positively impact racial and ethnic disparities given that lack of a usual source of care was a negative predictor of specialty care and chronic disease. However, this is not the complete solution in isolation. Over 90% of the African Americans in the sample reported a regular health care provider. This suggests a need for interventions targeting person-level barriers, such as self-efficacy and chronic disease knowledge. Since poor perceived health was associated with specialty care utilization and chronic disease status, efforts to provide knowledge could provide necessary knowledge about the chronic disease diagnosis. Increased knowledge, both provider and patient, will assist with patient orientation and provider/practice coordination. However, it is critical to not attribute negative attributes, such as nonadherent and noncompliant behavior, to patients while overlooking the impact of system deficiencies [45–48]. It could be more productive to organize practice systems and implement approaches to patient care that improve patient follow-through on treatment plans [49,50].

The disconnect between primary care providers and lack of specialty care use, especially in minorities with chronic diseases, suggest the need for system coordination. Electronic health records or EHRs to monitor and treat patients with chronic illness, often vulnerable, underserved populations (minority, rural) can assist in the coordination of care between primary and specialty care providers. Patients have reported dissatisfaction with specialty care due to long wait times and the lack of interpersonal patient-provider interaction. However, incorporating physician assistants (PAs) and nurse practitioners (NPs) in medical practices to improve access to services, reduce wait times, and improve quality of care could increase coordination and assist in patients efficiently navigating the health care system [49,50]. In particular for patients with chronic disease, a stepped approach to care is supported by the study findings. Stepped care provides a framework for achieving professional support for chronic illness that is cost-effective and is based on patients' observed response to treatment; simpler interventions are tried first, with more intensive interventions reserved for when a good outcome is not achieved [51,52]. These recommendations would allow for more coordinated care and ultimately improved outcomes for vulnerable populations. It also contributes to the growing body of literature that suggests that shared responsibility for primary and specialty care allow for optimal quality of care [7].

However, there are several limitations to the study, including potential bias related to self-report measures, the low response rate of those defined as non-White or non-African American Others, and the inability to probe for other factors that may potentially serve as barriers to specialty care. In addition, a longitudinal dataset may yield different findings about the use of specialty care and chronic disease status over time. The study findings provide further evidence for the continued exploration of the use of specialty care as it relates to health disparities.

Practice, research, and policy implications from the evidence are essential to advancing public health knowledge. Culturally and linguistically appropriate services and health behavior interventions are necessary for reducing the health disparities from the study’s findings. An economically disadvantaged population with limited English proficiency has difficulty accessing the health care system, especially for specialty care. Bilingual efforts to promote the use of specialty and methods to eliminate patient and system level barriers are needed. Culturally appropriate health promotion and behavior interventions are supported by the findings. African Americans reported higher rates of diabetes than the other groups in the sample. Efforts to identify and reduce geographically determinants or barriers to care should be explored in future research.

The increasing rigor of health disparities research calls for the use of nationally representative data with significant minority respondents. This study and others that include multivariate analysis allow for a thorough examination of existing health disparities and the factors associated with disparities in care.

## Figures and Tables

**Table 1.** Selected demographic characteristics by race/ethnicity.

**Variables**	**White**	**AA**	**Other**	**Total**

Unweighted observations	43.3% (1072)	32% (792)	24.7% (611)	2475

**Utilization of specialty care**				

Positive	55.2	49.2	44.0	50.6

Negative	44.8	50.8	56.0	49.5

**Gender**				
Male	32.7	31.1	41.1	34.2
Female	67.4	68.9	29.6	65.8
**Age**				
18–44	37.7	43.3	61.7	45.4
45–64	36.9	37.9	28.6	35.2
65+	25.4	18.8	9.7	19.4
Marital Status **				
Married	59.7	37.5	61.1	52.9
Not Married	26.9	31.8	14.7	25.5
Never Married	13.4	30.7	24.2	21.6
Employment Status **				
Work for wages	80.5	77.4	79.7	79.3
Not employed	19.5	22.6	20.3	20.7
Education Level **				
Less than HS diploma	13.4	16.5	8.5	13.2
HS graduate	29.3	32.2	20.6	28.1
Some college	26.2	28.0	22.3	25.8
College & Post-graduate	31.1	23.2	48.6	32.9
Geographic Location **				
Urban	81.3	90.4	93.6	87.3
Rural	18.7	9.6	6.4	12.7
Annual Income **				
<$25,000	72.6	59.7	78.2	69.9
$25,000–<$60,000	7.0	14.1	8.2	9.6
$60,000+	20.4	26.1	13.6	20.6
Nativity**				
Foreign-born	18.2	7.2	55.8	24.0
US-born	81.8	92.8	44.2	76.0
Language of Interview**				
English Interview	87.2	99.5	96.9	93.5
Non-English Interview	12.8	0.5	3.1	6.5
**Perceived health status**				
Positive	80.3	77.3	88.7	81.4
Negative	19.7	22.7	11.3	18.6
**Health Insurance Status**				
Yes, no interruptions	83.4	82.6	83.5	83.2
**Regular Healthcare Provider**				
Yes	91.2	90.5	88.4	90.3
No	8.8	9.5	11.6	9.7

Significantly different than white respondents;

* < 0.05,

** < 0.01.

**Table 2.** Chronic disease characteristics by race and ethnicity.

**Variables**	**White**	**AA**	**Other**	**Total**

**Chronic Diseases**				
Chronic diseases	46.6	55.8	32.2	46.0
Diabetes	11.4	17.3	9.5	12.8
High blood pressure	32.7	42.8	21.4	33.1
Lung diseases	14.0	17.6	8.2	13.7
Heart diseases	10.6	10.1	3.9	8.8

**Table 3.** Factors Associated with Specialty Care Use, Weighted Sample 29,454,000.

**Variables**	**Full Model**		**Final Reduced Model**	
	**OR**	**95% CI**	**AOR**	**95% CI**

**Demographic Characteristics**				
**Sex (ref = male)**				
Female	1.10	0.86 1.32	1.1	0.87 1.31
**Race & Ethnicity (ref = White)**				
African American	0.70	0.52 0.81	0.8	0.67 0.99
Other	0.68	0.52 0.88	0.7	0.60 0.92
**Age (ref = 18–44)**				
45–64	1.50	1.17 1.91	1.8	1.44 2.23
65+	1.41	1.01 1.96	1.8	1.35 2.31
**Education (ref = HS grad)**				
Less than HS diploma	0.71	0.49 1.04	-----	----- -----
Some college	1.21	0.92 1.58	-----	----- -----
College & Post-graduate	1.26	0.96 1.66	-----	----- -----
**Employment (Ref = Employed)**				
Not Employed	1.54	1.17 2.02	-----	----- -----
**Annual Income(Ref = $60,000+)**				
<$25,000	1.29	0.97 1.73	1.4	1.10 1.85
$25,000–<$60,000	1.28	0.84 1.95	1.30	0.86 1.94
**Language of Interview**				
Non-English (Ref = English)	0.41	0.24 0.69	-----	----- -----
**Chronic Dx Status (Ref = Positive)**	0.55	0.44 0.69	-----	----- -----
Chronic Dx Status Negative				
**Enabling variables**				
**Insurance status (Ref = Insured)**				
Health Ins coverage, No Rx	0.65	0.45 0.95	0.6	0.42 0.85
No Health Ins Coverage, only Rx	0.50	0.19 1.27	0.6	0.21 1.56
Uninsured	0.58	0.35 0.95	0.4	0.28 0.61
**Regular Health Care Provider**				
No Reg. Provider (Ref = Yes)	0.46	0.31 0.68	0.4	0.27 0.59
**Need Variables**				
**Perceived health status**				
Fair/poor (Ref = good/excellent)	1.93	1.43 2.6	1.7	1.33 2.23

OR = Odds Ratio; AOR = Adjusted Odds Ratio.

**Table 4.** Factors Associated with Chronic Disease Status, Weighted Sample 29,454,000.

**Variables**	**Full Model**		**Final Reduced Model**	
	**OR**	**95% CI**	**AOR**	**95% CI**

**Demographic Characteristics**				
**Sex (ref = male)**				
Female	0.84	0.67 1.06	0.87	0.70 1.09
**Race & Ethnicity (ref = White)**				
African American	1.58	1.24 2.01	1.66	1.33 2.08
Other	1.02	0.77 1.34	1.10	0.86 1.42
**Age (ref = 18**–**44)**				
45–64	3.86	3.0 5.0	3.90	3.07 4.95
65+	8.17	5.79 11.54	8.45	6.17 11.56
**Education (ref = HS grad)**				
Less than HS diploma	1.51	1.01 2.26	-----	----- -----
Some college	1.35	1.00 1.81	-----	----- -----
College & Post-graduate	0.96	0.72 1.29	-----	----- -----
**Nativity**				
Foreign-born (referent = US born)	0.66	0.48 0.90	0.55	0.42 0.72
**Annual Income(Ref = $60,000+)**				
<$25,000	0.81	0.59 1.10	0.76	0.57 1.02
$25,000–<$60,000	1.15	0.71 1.87	1.16	0.72 1.84
**Language of Interview**				
Non-English (Ref = English)	0.54	0.31 0.93	-----	----- -----
**Enabling variables**				
Health Ins coverage, No Rx	0.59	0.40 0.88	0.62	0.42 0.91
No Health Ins Coverage, only Rx	1.18	0.33 4.21	1.25	0.37 4.23
Uninsured	0.63	0.36 1.11	0.76	0.50 1.16
**Regular Health Care Provider**				
No Reg. Provider (Ref = Yes)	0.46	0.31 0.69	0.47	0.31 0.70
**Need Variables**				
**Perceived health status**				
Fair/poor (Ref = good/excellent)	3.34	2.43 4.59	3.21	2.38 4.34

OR = Odds Ratio; AOR = Adjusted Odds Ratio.
